# Parasocial relationships on YouTube reduce prejudice towards mental health issues

**DOI:** 10.1038/s41598-022-17487-3

**Published:** 2022-10-04

**Authors:** Shaaba Lotun, Veronica M. Lamarche, Spyridon Samothrakis, Gillian M. Sandstrom, Ana Matran-Fernandez

**Affiliations:** 1 Department of Psychology, University of Essex, Colchester, CO4 3SQ UK; 2 Institute for Analytics and Data Science, University of Essex, Colchester, CO4 3SQ UK; 3 Brain-Computer Interfaces and Neural Engineering Lab, School of Computer Science and Electronic Engineering, University of Essex, Colchester, CO4 3SQ UK

**Keywords:** Human behaviour, Social behaviour

## Abstract

Intergroup contact has long been established as a way to reduce prejudice among society, but in-person interventions can be resource intensive and limited in reach. Parasocial relationships (PSRs) might navigate these problems by reaching large audiences with minimal resources and have been shown to help reduce prejudice in an extended version of contact theory. However, previous studies have shown inconsistent success. We assessed whether parasocial interventions reduce prejudice towards people with mental health issues by first creating a new PSR with a YouTube creator disclosing their experiences with borderline personality disorder. Our intervention successfully reduced explicit prejudice and intergroup anxiety. We corroborated these effects through causal analyses, where lower prejudice levels were mediated by the strength of parasocial bond. Preliminary findings suggest that this lower prejudice is sustained over time. Our results support the parasocial contact hypothesis and provide an organic method to passively reduce prejudice on a large scale.

## Introduction

We constantly form connections with people who are unaware of our existence, and they influence how we behave. These one-sided psychological bonds are called parasocial relationships (PSRs)^1^. We often build them with real or fictional figures that we never directly interact with, such as television characters and celebrities. In these PSRs, we get to know targets who unidirectionally disclose information to masses of viewers, in ways that cannot be meaningfully returned. For fictional targets (e.g., book characters), reciprocation would be impossible. For non-fictional people (e.g., newscasters and celebrities), online technology provides unprecedented insights that make us perceive them as friends, even though they will never experience our self-disclosing as we experience theirs. Research on parasocial ties has shown that PSRs can satisfy our affective, behavioural, and cognitive needs similarly to traditional bidirectional friendships^2^: they make us feel less lonely^3^, influence our purchasing decisions^4^, and we even assimilate their body traits to the self^5^. However, challenging the boundaries of what parasocial ties are known to contribute, can the unidirectional disclosure from PSRs be harnessed for more substantial change, such as reducing societal prejudice?

Prejudice is a negative evaluation of others based on group membership^6^. Considering this intergroup attitude, ingroups are positively identified and outgroups are relatively devalued whenever degrees of differences exist. According to contact theory, mutual interpersonal contact can effectively reduce prejudice^7^, as disclosure increases perceived similarity to generate reciprocal trust^8^. For this reason, it seems less fathomable that unidirectional disclosure from a parasocial target could contribute to prejudice reduction. On the other hand, media has long played a role in shaping societal beliefs, including children’s shows like *Sesame Street*^9^. Despite beliefs being extremely resistant to change, media influence can broadcast norms, inspire an empathy that is generalisable to one’s real-world society^10^, and reduce social distance and avoidance of outgroups, by highlighting intergroup differences^11^.

In a non-randomised correlational study, increased parasocial interaction with gay sitcom characters predicted lower homosexual prejudice and an extended parasocial contact hypothesis was proposed, purporting that parasocial interaction may reduce prejudice in similar ways to contact theory^12^. Causality was supported in a similar study that replicated the context of heterosexual television characters, where parasocial interaction was found to reduce sexual prejudice^13^. Our research builds on these studies with an experimental design assessing parasocial intervention in the context of non-fictional social media. Further, causal analysis methods are used to assess whether PSR strength specifically is the mechanism operating this influence, supporting findings in traditional media contexts^13^. This contributes unique insights to the field of parasocial interventions, as although select studies have explored causal claims of parasocial contact^13^, most past research relies on correlations with pre-existing parasocial relationships, or only observes traditional media contexts, or compares prejudice reduction capabilities from different media formats (e.g., videos vs. magazines, or music videos vs. imagined contact)^11,14^. In this way, our study expands on the ‘what’ of prejudice reduction theory, and ventures into the ‘why’.

This is pertinent as, despite prejudice being one of the most active areas of inquiry in social psychology where the theoretical concepts are well established^7,15^, less is known about the mechanisms behind successful prejudice reduction in the world^16^. This is particularly true with its understanding of media contributions^17^, where parasocial prejudice interventions have demonstrated inconsistent success. *Harry Potter* excerpts improved child attitudes to homosexuality and immigration^18^, and *Shrek* promoted a higher understanding of stigma^19^, but *The Walking Dead* assimilation failed to change racist behaviour^20^, and a Rwandan radio show had no effect on prejudicial beliefs^17^. Such inconsistencies may be due to a lack of understanding of what exactly causes prejudice reduction, or perhaps indicate study limitations. Systemic reviews of prejudice studies found that from 1958 to 2008, only twelve studies evaluated prejudice reduction in a randomised field with non-student samples^14^, very few used implicit and explicit measures, and even fewer pre-registered or examined effects over time^21^. Furthermore, out of hundreds of intervention studies, only 11% tested causal prejudice effects in the real world and among adults^16^, limiting academic knowledge outside the laboratory.

Another notable literary gap is the context of parasocial interaction. Many past prejudice intervention studies have focused on fictional parasocial target interactions, such as television characters^12,13^. Whilst important, no studies to date have explored prejudice interventions with online creators, who are non-fictional, as parasocial targets. As past research has justified extending theoretical parasocial expectations from traditional media to online platforms such as YouTube^22^, it seems reasonable to explore whether prejudice interventions in traditional media contexts work equally as well across social media. As an increasingly prevalent medium, particularly among young adults^22^, millions of people watch creators share their lives and opinions on social media, such as through YouTube videos. Unlike fictional characters or less-relatable celebrities, creators present a preeminent resource for nuanced outgroup representation, as the accounts of real people contrast the often-sensationalised outgroup portrayal in traditional media^23^. LGBT+ people, for example, are mostly absent or portrayed as deviant on television^24^, but a gay creator talking about their personal experiences online might better reflect the real-world outgroup lens. In lieu of in-person contact, such increasingly accessible outgroup portrayal may de-stigmatise identities and reduce prejudice more effectively^25^.

Using real creators as parasocial targets and real stimuli from YouTube, the present study aims to overcome the limitations above through an intervention that functions in laboratory settings and the real world alike. We measured prejudice towards a highly stigmatised issue (namely, mental health) across implicit, explicit, and behavioural dimensions, in a randomised adult sample, whilst evaluating whether the changes in prejudice brought upon by the intervention were maintained across time. Using a parasocial version of the original ‘fast friends’ paradigm^26^ (which we term the Parasocial Fast Friends Paradigm, PFFP), we created new PSRs between our participants and a parasocial target previously unknown to them and explored whether parasocial strength can reduce prejudice towards people with mental health issues.

## Methods

### Participants

A total of 557 participants were recruited using the online participant pool *Prolific*, of which 333 completed the experiment. To prime optimal understanding and effectiveness of the stimuli used in this study, only those who satisfied the following four criteria were invited to take part: not knowing the two creators featured in the stimuli; being between 18–35 years of age (a similar audience age of the creators); having English as a first language; and not having experienced significant mental health issues such as Borderline Personality Disorder (BPD) or close contacts who have such experience.

**Figure 1:**
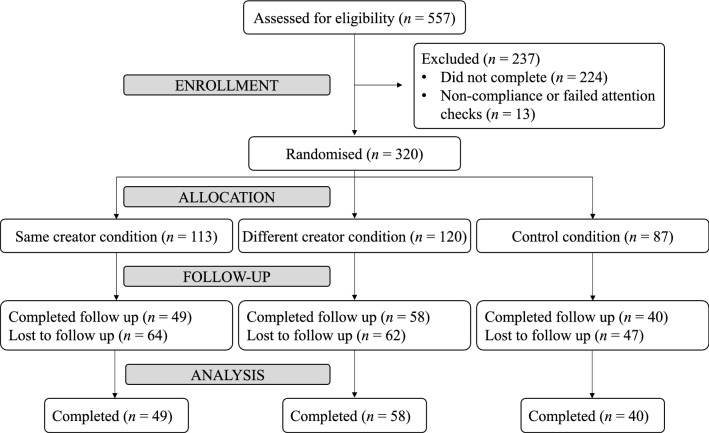
Diagram of participant flow.

The subjects’ inclusion and exclusion in this analysis are shown in Fig. 1. Out of the 333 participants that completed the experiment, 13 responses were removed due to non-compliance with Implicit Association Test (IAT) instructions or failing more than one attention check (see Procedure section below), resulting in a final sample of 320 usable responses (191 identified as female, 126 as male, and 3 as non-binary; mean age = 26±4.9 years old). All participants who completed the experiment were financially compensated.

In addition, those who completed an optional follow-up survey a week after the initial task were entered into a prize draw for retail vouchers. Of the final 320 responses, 147 participants voluntarily took part in the follow-up survey.

This study was pre-registered on the Open Science Framework (https://osf.io/qkda8). The experiment received ethical approval by the Ethics Committee of the University of Essex in February 2021 (ETH2021-0937) and was performed in accordance with relevant guidelines and regulations. Participants were given ample opportunity to ask questions and informed consent was obtained prior to data collection, which took place between April and May 2021.

### Experimental protocol

**Figure 2:**
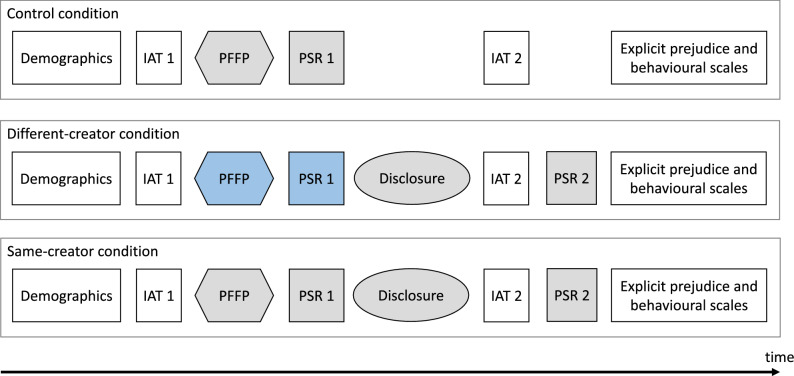
Experimental protocol. The items for which Creator A is featured are filled in gray, the ones that refer to Creator B are in blue, and the transparent blocks are common across the three conditions.

The order and components of the experiment are shown in Fig. 2. Participants were randomly assigned to either the same-creator condition ($n = 113$), different-creator condition ($n = 120$), or control condition ($n = 87$).

After reporting demographic information, all participants completed an IAT designed to measure implicit attitudes towards people with mental health issues. This was followed by a relationship-building video, in which Creator A (for participants in the same-creator and control conditions) or Creator B (for participants in the different-creator condition) answered a series of increasingly intimate “get to know me” questions as per the parasocial Fast Friends Paradigm^26^ (referred to as PFFP in Fig. 2).

In the second video, Creator A talked about her experiences with mental health, specifically BPD. Participants in the control condition watched the relationship-building video from Creator A only. Following attention checks (see Video stimuli section), participants completed a scale to measure PSR strength with the featured creator. For the same-creator and different-creator conditions, participants then watched the disclosure stimulus featuring Creator A talking about her experiences with BPD and dispelling common myths. Following further attention checks and PSR measures (same-creator and different-creator conditions only), all participants completed the IAT again. Since participants in the control condition did not watch the second video, this allowed us to control for changes in implicit prejudice levels being due to task familiarity with the IAT rather than actual lowering of prejudice. Finally, participants filled the explicit prejudice scales and the questions about behavioural attitudes towards people with mental health issues. The order in which the scales and items (within a scale) were presented was randomised where possible.

Participants were not able to pause, replay or skip the stimuli during watching.

In the follow-up survey, which was sent to participants one week later, participants were asked again to fill the explicit and behavioural prejudice measures (not shown in Fig. 2).

#### Hypotheses

We hypothesised that the levels of implicit and explicit prejudice for participants in the same- and different-creator conditions would decrease, but they would remain constant for the control condition (*Hypothesis 1*). Moreover, those in the same-creator condition would show lower levels of implicit, explicit and behavioural prejudice, and intergroup anxiety, than the different-creator condition, which in turn, will show less prejudice than the control condition (*Hypothesis 2*).

With regards to PSR, we hypothesised that those in the same-creator condition would experience greater parasocial strength towards Creator A than participants in the different-creator condition after the intervention (*Hypothesis 3*) and that greater PSR towards the disclosing creator would result in lower levels of explicit prejudice after the intervention (*Hypothesis 4*).

Finally, with respect to the long-term effects of the intervention, we hypothesised that lower prejudice levels would be maintained over time, and those exposed to the disclosure stimuli (i.e., both in the same- and different-creator conditions) would still report lower prejudice levels after one week than those in the control condition (*Hypothesis 5*).

### Video stimuli

#### Relationship-building videos

The Fast Friends Paradigm^26^ successfully creates a new relationship between two strangers meeting in person in as little as 9 minutes. In the original version, pairs of people take turns to answer 3 sets of personal questions, each set increasing in intimacy, that prompt self-disclosure about general topics, such as upbringing, memories, and values. We used the Parasocial Fast Friends Paradigm (PFFP)^27^ to create a PSR between the viewer and a creator. In the PFFP, the viewer watches a 9-min video in which the creator answers the questions from the original fast friends paradigm facing the camera. The PFFP has been successful at forming parasocial relationships with viewers who had no prior knowledge of the creator^27^.

In our experiment, two different creators (which we call Creator A and Creator B) recorded themselves answering the same set of questions in the same order, and with the same filming and editing set ups to avoid undue variance. In this way, we had two versions of the relationship-building stimuli.

To check that participants had paid attention to the video, they were asked 2 questions about the content of the video and one about the appearance of the creator.

#### Self-disclosure stimuli

Only one version of this stimulus was created, in which Creator A discusses her personal journey with BPD and how the public perceives the disorder, and answers some of the most-searched questions about BPD from her point of view. To create this video, we chose organic video content from this creator’s Youtube channel. The edited video was 17 minutes long, and featured minimal editing and a similar filming set up as the PFFP stimuli.

To check that participants had paid attention to the video, they were asked one question about its content.

Participants that responded wrongly to at least one of the attention check questions (two in the relationship-building stage and one in the disclosure stage) were disqualified from the study.

### Measures

#### Prejudice

We used the following four measures to assess the prejudice levels of the participants.

##### Implicit prejudice

We built our IAT using *iatgen*^28^, based on the publicly available mental health prejudice IAT^29^. The IAT is a computer-based response latency test that gauges participant automaticity in associating safe or unsafe evaluative concepts (e.g., ‘safe’ vs. ‘unsafe’, ‘harmless’ vs. ‘dangerous’) with social group categories of ‘people with mental health issues’ (e.g., borderline personality disorder and schizophrenia) and ‘people with physical health issues’ (e.g., diabetes and multiple sclerosis). Our IAT consisted of 7 blocks as shown in Table 1, including practice trials (blocks 1–2) to allow participants to familiarise themselves with the task.

**Table 1:** Sequence of trial blocks for the mental health prejudice IAT.

Block	No. of trials	Function	Items assigned to left-key	Items assigned to right-key
1	20	Practice	Mental health disorders	Physical health disorders
2	20	Practice	Safe words	Unsafe words
3	20	Practice	Safe words + mental health disorders	Unsafe words + physical health disorders
4	40	Test	Safe words + mental health disorders	Unsafe words + physical health disorders
5	20	Practice	Physical health disorders	Mental health disorders
6	20	Practice	Safe words + physical health disorders	Unsafe words + mental health disorders
7	40	Test	Safe words + physical health disorders	Unsafe words + mental health disorders

In each trial, participants were shown a word from the mental health, physical health, safe, or unsafe categories, and asked to categorise it using two different key presses. If participants made an error, they were asked to correct the mistake before displaying the next word. Differences were calculated in how quickly participants associated individual words to grouped categories and were converted into a *d* score (with values ranging between − 1 and 1), where negative *d* scores represent more bias towards mental health issues (with respect to physical health issues) and positive *d* scores represent more bias to physical health issues. More specifically, when calculating *d* scores, individual responses over 10 s were deleted, as well as data from participants that had more than 10% of their responses faster than 300 ms. The *d* score for each participant was calculated by dividing the within-subject difference between the compatible (mental health with safe concepts) and incompatible (mental health with unsafe concepts) block means, by a pooled standard deviation, and averaging the resulting scores^28^. This was done for each participant and IAT task (once before exposure to intervention stimuli and once post-exposure; shown in Fig. 2 as *IAT 1* and *IAT 2*, respectively).

##### Explicit prejudice

We used a shortened version of the Prejudice Towards People With Mental Illness Scale^30^ with a Likert-scale ranging from strongly disagree (1) to strongly agree (7). For this study, only two of the four original subscales were used: malevolence and fear and avoidance. Item wording was amended to refer to “people with BPD”. As pre-registered, since the reliability for both subscales was higher than our pre-defined threshold of 0.5 ($\alpha _{fear} = 0.69$, $\alpha _{malevolence} = 0.81$), we combined them into an overall measure of explicit prejudice ($\alpha _{combined} =0.82$).

##### Intergroup anxiety

We used a 7-point scale (1 = strongly disagree, 7 = strongly agree) of the 11-item Intergroup Anxiety Toward Muslim Scale^31^. Item wording was adapted to refer to people with BPD instead of Muslims, and the items were averaged to create an overall measure of intergroup anxiety ($\alpha _{anxiety}$ = 0.92).

##### Behavioural measures

After watching the disclosure video, participants were asked two behavioural questions^14^ on whether they would be willing to volunteer with an organisation to help those with BPD, and if they would like to receive information about BPD campaigns. Both items were examined separately in analyses.

Additionally, in the follow-up survey, participants were asked three further questions: if they had thought about people with BPD; if they had actively contributed towards raising awareness or protecting the rights of people with mental health issues (e.g., having positive or educational conversations with others about it, or donating time or money towards initiatives dedicated to the cause); and if they had actively done anything to contribute against raising awareness or protecting the rights of people with mental health issues (e.g., having negative conversations with others about it, or donating time or money towards initiatives that do not support mental health). The final two items were combined and coded on a scale of 1–5 (1 = actively contributing towards anti-support measures, 2 = discussing anti-support, 3 = no action, 4 = discussing support, 5 = actively contributing towards support measures).

#### Parasocial relationships

A 16-item version of the Celebrity Personal Parasocial Interaction Scale^32^ was used on a 7-point scale (1 = strongly disagree, 7 = strongly agree). Four items were removed due to their reliance on a pre-existing PSR with the featured target. All items were averaged to create an overall measure for each of the PSR surveys ($\alpha _{PSR1}$ = 0.91, $\alpha _{PSR2}$ = 0.90).

## Results

### Implicit prejudice

**Figure 3:**
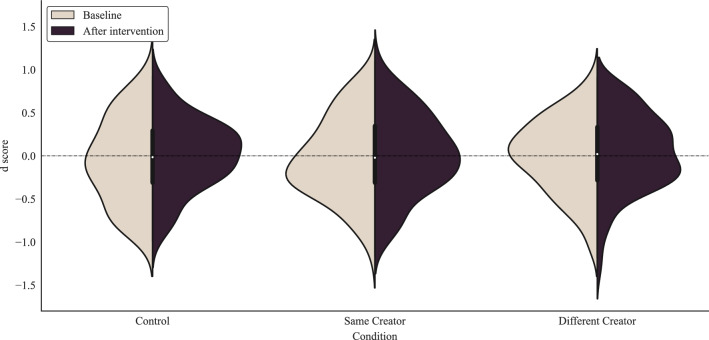
Implicit prejudice *d* scores (as measured by the IAT) pre- and post-intervention for each condition. Positive (resp. negative) values of *d* represent less (resp. more) prejudice towards people with mental health issues (w.r.t. physical health issues).

We had postulated that implicit prejudice would decrease for participants in the same- and different-creator conditions, but remain approximately constant for those in the control condition (*Hypothesis 1*). To test for changes in implicit prejudice following the disclosure videos, *d* scores were created from each participant IAT responses both pre- and post-intervention. The distributions of *d* scores are shown in Fig. 3 for each condition. Due to the non-Gaussian distribution of the data, we used a non-parametric paired Wilcoxon signed-rank test to test for differences in the implicit prejudice *d* scores from before and after the disclosure stimuli within each condition. Against our prediction, no significant differences were found between pre- and post-disclosure *d* scores for any of the three conditions. Average values and *p* values are reported in Table 2.

**Table 2:** Descriptive statistics on our sample (mean ± standard deviation), by condition, across measures of prejudice and PSR.

		Control condition	Same-creator condition	Different-creator condition
Implicit	IAT 1 *d* scores	− 0.06 ± 0.5	− 0.03 ± 0.47	− 0.0 ± 0.43
	IAT 2 *d* scores	0.01 ± 0.41	0.02 ± 0.47	0.04 ± 0.44
	Wilcoxon test	$W=1607$, $p=.19$	$W=2751$, $p=.18$	$W=3361, p=.48$
Explicit	Fear and avoidance	3.34 ± 0.79	2.99 ± 0.73	3.05 ± 0.76
	Malevolence	2.17 ± 0.88	2.07 ± 0.77	2.09 ± 0.8
	Explicit prejudice (combined)	2.76 ± 0.69	2.53 ± 0.64	2.57 ± 0.67
**Intergroup anxiety**		2.99 ± 1.13	2.66 ± 1.2	2.74 ± 1.07
Behavioural	Desire to volunteer	4.93 ± 5.26	3.95 ± 4.86	4.46 ± 4.7
	Desire to learn more	0.36 ± 0.48	0.33 ± 0.47	0.30 ± 0.46
PSR	PSR 1	3.71 ± 1.1	3.65 ± 1.09	3.65 ± 1.07
	PSR 2	N/A	3.87 ± 1.14	3.91 ± 0.94

### Explicit prejudice

To test for differences in explicit prejudice, analyses of variance (ANOVA) with planned contrasts were used for the explicit prejudice measures.

**Figure 4:**
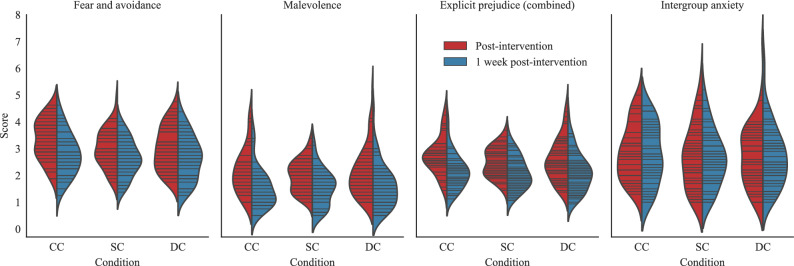
Explicit prejudice scores per condition, for each of the explicit measures, after the intervention vs. at the 1-week follow-up survey. The lines inside the plots represent the underlying data points for each of the distributions.

Using the combined explicit prejudice measure, a significant effect was found when comparing between conditions ($F(2, 317) = 3.093$, $p = .047$). Planned contrasts showed that participants in the same-creator condition had significantly lower explicit prejudice after watching the disclosing stimuli than those in the control condition ($M_{SC} = 2.53$; $M_{CC}=2.76$; $t(317) = -2.362$, $p = .019$). The different-creator condition also had significantly lower explicit prejudice than the control condition ($M_{DC} = 2.57$; $t(317) = -1.990$, $p = .047$), as stated in *Hypothesis 2*. However, against our prediction, the scores for the same- and different-creator conditions did not significantly differ from each other ($t(317) = -.432$, $p = .666$). The distributions of scores for the explicit prejudice scales are labelled as “Post-intervention” in Fig. 4.

The same pattern between conditions was found within the fear and avoidance sub-scale ($F(2, 317) = 5.689$, $p = .004$), where participants in the same-creator condition had significantly lower fear and avoidance scores after watching the disclosing stimuli ($M_{SC}=2.99$) than those in the control condition ($M_{CC} = 3.34$; $t(317) = -3.196$, $p = .002$). Participants in the different-creator condition also had significantly lower scores than those in the control condition ($M_{DC} = 3.05$; $t(317) = -2.714$, $p = .007$), but the same- and different-creator conditions did not significantly differ from each other ($t(317) = -.562$, $p = .575$).

Finally, no significant differences between conditions were found within the sub-scale of malevolence or any of its planned contrasts ($F(2, 317) = .460$, $p = .632$), although the malevolence values were already low (an average score of 2.17 out of 7 in the control condition, vs. the average value of 3.34 out of 7 for the fear and avoidance subscale), so changes here would not necessarily provide substantial prosocial value.

### Intergroup anxiety

A planned contrast ANOVA was used to compare intergroup anxiety between conditions ($F(2, 317) = 2.27$, $p = .105$). Those in the same-creator condition had significantly lower intergroup anxiety ($M_{SC}=2.66$) than participants assigned to the control condition ($M_{CC}=2.99$; $t(317) = -2.075$, $p = .039$). As in explicit prejudice, participants in the same- and different-creator ($M_{DC} = 2.73$) conditions did not significantly differ from each other ($t(317) = -.555$, $p = .579$), nor did the scores from participants from the different creator condition with respect to the control condition ($t(317)=-1.585$, $p=.114$). The distributions of scores for the three conditions are shown in Fig. 4.

### Behavioural measures

Finally, a planned contrast ANOVA was used to assess the behavioural items in our survey. Nor the behavioural items for volunteering ($F(2, 317) = .997$, $p = .370$) or receiving information, ($F(2, 317) = .364$, $p = .695$) differed significantly across the 3 conditions (see Table 2 for descriptive statistics).

### Interim discussion

To summarise prejudice results, no significant differences were found in implicit prejudice or in the two behavioural measures directly after watching the disclosing video. However, measures of explicit prejudice, and specifically intergroup anxiety and the subscale of fear and avoidance, showed that those exposed to the disclosure video (both in the same- and the different-creator conditions) had lower prejudice and intergroup anxiety levels than those who were not. While these explicit prejudice results support our initial hypotheses, prejudice levels between the same- and different-creator conditions did not differ from each other. Further exploration of parasocial relationship strength between these conditions was therefore conducted.

### Influence of parasocial relationship strength on prejudice

We had predicted (*Hypothesis 3*) that parasocial relationship strength towards Creator A (i.e., the disclosing target) after the intervention (i.e., PSR 2) would be greater for participants assigned to the same-creator condition than those in the different-creator condition. Before testing this hypothesis, we ran a control check to ensure that the individual differences between the two creators did not influence PSR strength. A Kruskal-Wallis test compared PSR 1 strength across the three conditions after watching the relationship-building stimuli. No significant differences were found between participants from the same-creator condition, the control condition (both of whom watched the Parasocial Fast Friends Paradigm video featuring Creator A), and the different-creator condition (who watched the PFFP video featuring Creator B; $H=.134$, $p=.935$).

Contrary to *Hypothesis 3*, participants that watched Creator A’s relationship-building stimuli in the same-creator condition did not produce significantly greater PSR2 strength towards them after the disclosure about mental health, when compared to the different-creator condition (which watched the relationship-building video from Creator B and then the disclosure video from Creator A (Mann-Whitney $U=6591, p=0.32$).

Even though our experimental protocol failed at manipulating PSR 2 strength across conditions, we found a significant correlation between PSR 2 strength and the combined explicit prejudice measure that corroborated our *Hypothesis 4*, which stated that greater PSR to the disclosing creator would result in lower prejudice levels after disclosure (Pearson’s $\rho = -.296$, $p < .001$). PSR 2 also correlated significantly with the explicit sub-scales of fear and avoidance (Pearson’s $\rho = -.274$, $p < .001$) and malevolence ($\rho = -.241$, $p < .001$).

We were also interested in understanding whether the disclosure of BPD would strengthen or weaken the parasocial relationship created between participants in the same-creator condition and Creator A. A paired Wilcoxon test comparing PSR 1 and PSR 2 values for this group found that PSR strength increased after the disclosure video ($W=1695$, $p<.001$).

Finally, a means comparison was run to compare whether the relationship-building stimuli alone (where either creator spoke about themselves broadly using the Parasocial Fast Friends paradigm) or disclosing stimuli alone (where Creator A spoke about her BPD experiences) were more effective at creating PSR strength. A Mann–Whitney U test revealed that PSR 2 from the different-creator condition (following the disclosure stimuli alone, $M_{DC} = 3.91$) was significantly higher than PSR 1 from the same-creator and control conditions (following the relationship-building stimuli alone, $M_{SC,CC} = 3.68$; $U=10398, p=.023$).

To summarise PSR analyses, priming participants with the relationship-building stimuli before disclosure did not result in greater PSR strength than the disclosure stimuli alone, so the condition manipulation within the present study did not produce the expected results. If PSR strength mediates prejudice reduction, this manipulation failure is a possible explanation for why no significant differences were found between the same- and different-creator groups for explicit measures of prejudice or intergroup anxiety. In spite of this, PSR strength across conditions was significantly correlated with explicit prejudice, confirming our *Hypothesis 4*. Moreover, disclosure of BPD increased the strength of the PSR towards the disclosing creator for participants in the same-creator group. Lastly, the disclosure video on its own resulted in greater PSR strength than when either creator spoke broadly about themselves using the PFFP.

#### Causal analysis

Alongside pre-registered analyses, we performed a causal inference analysis to confirm that the significant changes found in explicit measures of prejudice were a result of experimental manipulation, and not extraneous confounding variables. The analysis was performed using *DoWhy*^33^.

**Figure 5:**
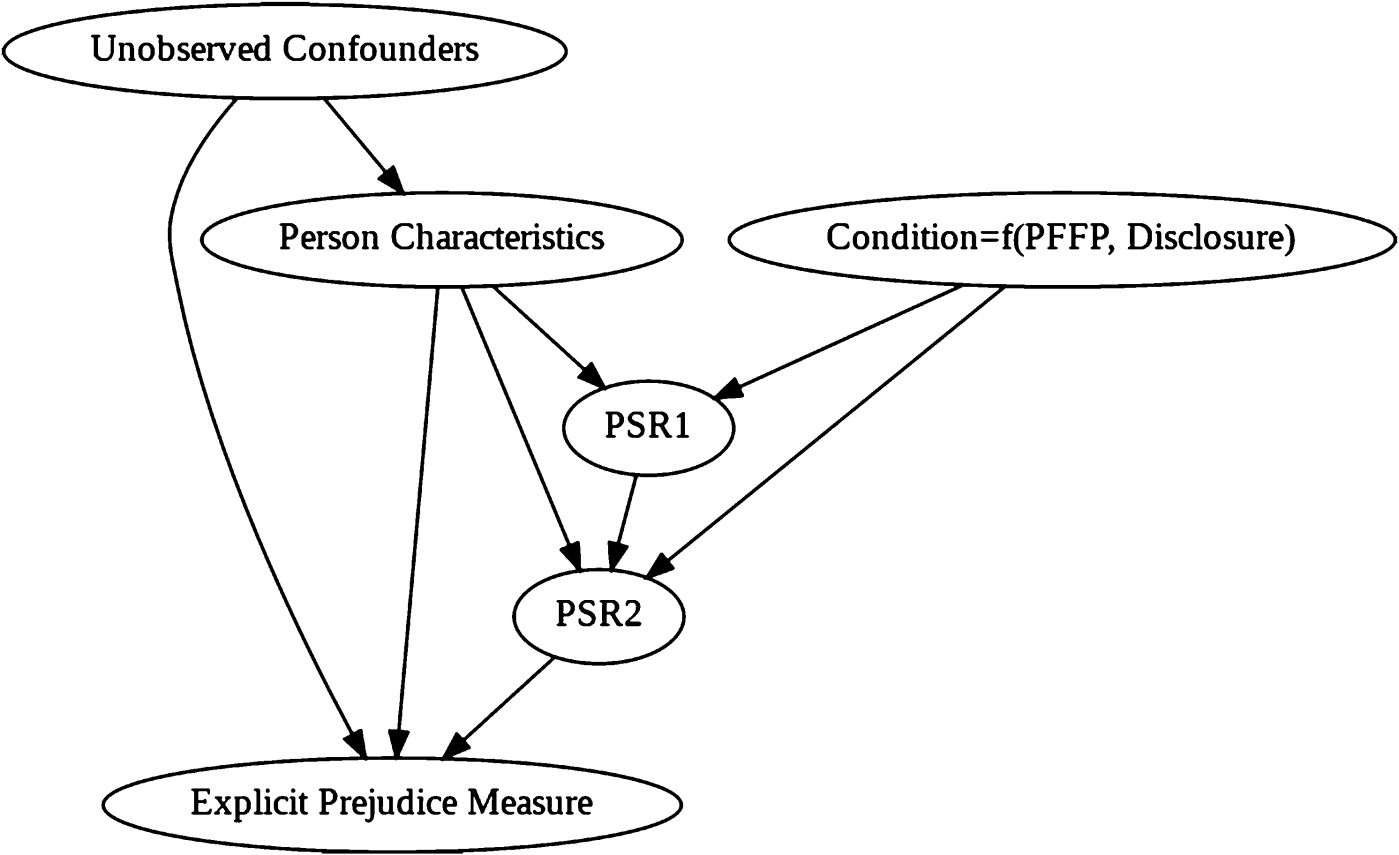
Causal graph for explicit prejudice measures.

We first constructed a causal graphical model based on our assumptions about the mechanisms for prejudice change in our experiment. This model is shown in Fig. 5. Namely, PSR 1 and PSR 2 are affected by each individual’s characteristics, as well as the experimental group to which they were assigned (“Condition”). The effect of the intervention on explicit prejudice is mediated by parasocial relationship strength (as well as the individual’s characteristics). “Condition” is the product of the identity of the creator in the PFFP and whether or not the participant watched the disclosure video. Note that, for participants in the control condition, PSR 2 is the same as PSR 1.

Five targeted confounders were considered: age, gender, ethnicity, sexual orientation, and proximity to loved ones with mental illness (summarised as “Person characteristics” in Fig. 5). Unobserved confounders were also considered for unrecorded individual differences. The treatment variable (“Condition” in Fig. 5) was used to compare the three groups separately for each explicit prejudice measure.

To estimate causal effects, we calculated the Average Treatment Effect (ATE), which measures the expected change to an outcome variable based on the group/condition. ATE estimates were generated using inverse propensity score weighting to account for possible imbalances in the confounders across the three experimental groups. The value of the ATE represents the average expected change in explicit prejudice (with respect to the baseline group) as a result of the intervention. We performed pairwise comparisons between the three groups. Our results are summarised in Table 3.

**Table 3:** ATE for explicit prejudice measures and intergroup anxiety, and results from different refutation methods. $p>.05$ in the refutation methods supports the validity of the effects found.

Groups	Outcome	ATE	Refutation methods		
			Random common cause	Data subset refuter	Placebo treatment
CC vs. DC	Fear and avoidance	${\textbf {-.26, p<.01, CI=[-42, -.07]}}$	− .27	− .26 (p=.41)	− .01 (p=.49)
	Malevolence	− .07, p=.26, CI=[− .25, .11]	–	–	–
	Explicit prejudice (combined)	**− .17, p=.04, CI=[− .31, − .01]**	− .16	− .16 (p=.46)	− .01 (p=.47)
	Intergroup anxiety	− .22, p=.08, CI=[− .48, .06]	–	–	–
CC vs. SC	Fear and avoidance	**− .27, p=.003, CI=[− .44, − .06]**	− .28	− .26 (p=.47)	.01 (p=.46)
	Malevolence	− .07, p=.25, CI=[− .26, .14]	–	–	–
	Explicit prejudice (combined)	**− .17, p=.036, CI=[− .31, .04]**	− .17	− .17 (p=.46)	.02 (p=.42)
	Intergroup anxiety	**− .27, p=.047, CI=[− .55, .09]**	− .26	− .26 (p=.49)	− .01 (p=.43)
SC vs. DC	Fear and avoidance	− .03, p=.38, CI=[− .16, .15]	–	–	–
	Malevolence	.01, p=.48, CI=[− .17, .16]	–	–	–
	Explicit prejudice (combined)	− .01, p=.46, CI=[− .13, .11]	–	–	–
	Intergroup anxiety	− .04, p=.42, CI=[− .26, .20]	–	–	–

Finally, in cases where a causal effect was found, we used three refutation methods to asses its robustness. The first refutation method added a random common cause to the dataset. In the second method, a data subset refuter bootstraps the dataset to replicate the analyses using different subsets of data. The third refutation method consists of repeating the analysis after randomly permutating the variable that indicates to which group an individual belongs, and is equivalent to having a placebo treatment. For the first two methods, the estimated ATE is not expected to change if the causal effects are robust. For the placebo method, the ATE is expected to be close to zero.

Overall, the results in Table 3 are consistent with those from our previous sections. Robust causal effects were found for the fear and avoidance subscale for the different- and same-creator groups with respect to the control group, showing that the disclosure video had a significant effect on the prejudice levels. Significant causal effects were also found for the combined explicit prejudice scale, but, as expected, not for the malevolence subscale.

When estimating causal effects for the intergroup anxiety measure across groups, we found that the reduction in prejudice found in the different creator condition was not significant with respect to the control group. However, the effect was robust for participants in the same creator group (vs. the control group).

As in previous analyses, no causal effects were found between belonging to the same vs. different-creator conditions. Considering that our manipulation failed to create higher PSR2 values for participants in the same-creator group than for those in the different-creator group, the lack of significant causal effects here is not surprising.

### Long-term prejudice reduction

Our final hypothesis (*Hypothesis 5*) stated that after one week, participants from the same- and different-creator conditions would still show lower prejudice levels (measured by the explicit prejudice and behavioural scales) than participants in the control condition. Summary statistics for explicit and behavioural prejudice measures directly after the intervention and for the 1-week follow-up survey are reported in Table 4. Power analyses carried out on *GPower*^34^, indicated that a minimum of 246 participants across the three groups were required to conduct a mean comparison across the conditions to detect a small effect size. Whilst the longitudinal sample did not meet this requirement (only 147 participants completed the follow-up survey; $n_{SC} = 49$, $n_{DC}=58$, $n_{CC}=40$), power analyses were satisfied for conducting Wilcoxon paired tests within conditions, comparing prejudice levels directly after watching the stimuli, and one week later. We did not find significant differences between post-intervention and 1-week follow-up surveys (after Bonferroni correction) for the combined explicit prejudice measure (corrected $p_{CC}=0.8$, $p_{SC}=1$, $p_{DC}=1$), nor for the the fear and avoidance subscale (corrected $p_{CC}=0.26$, $p_{SC}=1$, $p_{DC}=1$), the malevolence subscale (corrected $p_{CC}=0.3$, $p_{SC}=1$, $p_{DC}=1$), or intergroup anxiety (corrected $p_{CC}=1$, $p_{SC}=1$, $p_{DC}=1$).

**Table 4:** Descriptive statistics on our sample, at the end of the experiment and at the 1-week follow-up, per condition, for explicit and behavioural prejudice measures.

		CC		SC		DC	
		After intervention	Follow-up	After intervention	Follow-up	After intervention	Follow-up
Explicit	Fear and avoidance	3.32 ± 0.77	2.79 ± 0.80	3.00 ± 0.72	2.65 ± 0.69	3.0 ± 0.82	2.64 ± 0.84
	Malevolence	2.15 ± 0.86	1.52 ± 0.75	1.97 ± 0.59	1.54 ± 0.68	2.11 ± 0.93	1.60 ± 0.82
	Explicit prejudice (combined)	2.73 ± 0.69	2.15 ± 0.66	2.49 ± 0.55	2.09 ± 0.60	2.55 ± 0.79	2.12 ± 0.73
Intergroup anxiety		2.87 ± 1.04	2.78 ± 1.12	2.7 ± 1.21	2.63 ± 1.06	2.75 ± 1.18	2.71 ± 1.17
Behavioural	Thought	–	0.52 ± 0.51	–	0.71 ± 0.46	–	0.61 ± 0.49
	Contributed	–	0.22 ± 0.42	–	0.41 ± 0.50	–	0.26 ± 0.44

As for the behavioural items in the 1- week follow-up survey, participants were asked if they had thought about people with mental health issues such as BPD in the past week. While we recognise sample size requirements were not met for analysis of variance, an exploratory ANOVA found that those in the same-creator condition had thought about people with BPD more than those in the different-creator condition, which in turn had more thoughts than the control condition. Whilst none of these planned contrasts were significant ($F(2, 142) = 1.716$, $p=.183$), the comparison between the same-creator and control conditions was close to significance ($t(142) = 1.833$, $p = .069$), and may reach significance with an adequate sample size.

Similarly, when asking participants if in the last week they had thought specifically about helping those with mental health issues such as BPD, an exploratory ANOVA did not reach significance ($F(2, 143) = 2.096$, $p=.127$). Those in the same-creator condition had thought about taking prosocial action significantly more than those in the different-creator and control conditions combined ($t(143) = 2.038$, $p = .043$), although again this is only a suggestive finding due to the small sample size.

One final behaviour measure was coded, where participants were asked whether they had actively contributed towards (e.g., by having positive and educational conversations or donating time or money towards mental health initiatives), or against (e.g., by having negative conversations about mental health or donating time or money to initiatives that disregard mental health) people with mental health issues. None of the participants that filled the follow-up survey reported actively acting *against* people with mental health issues. 15 participants (10.2%) contributed towards people with mental health issues (7 from the same-creator condition, 5 from the different-creator condition, and 3 from the control condition). Actions reported by participants in support of mental health issues included having educational conversations about mental health with loved ones, further educating themselves through enrolling in courses, signing petitions, and donating and hosting fundraisers for mental health charities.

## Discussion

Due to their unidirectional quality, many people overlook the ability of PSRs to do anything more than reduce loneliness, entertain, and advertise products^3,35^. Building on the parasocial contact hypothesis, our research suggests there is more to be gained. The PSRs we passively form in everyday activities such as using social media, can help us develop as less prejudiced people. More specifically, our findings suggest that the PSR strength we develop with non-fictional disclosing targets can result in lower explicit prejudice and intergroup anxiety.

In this study, participants formed PSRs with a parasocial target who disclosed their experiences with BPD. Some participants formed this PSR before disclosure, others did not, and we measured participant prejudice levels towards people with mental health issues across implicit, explicit, and behavioural dimensions. Contrary to prediction, implicit prejudice levels were not affected by this prejudice intervention, but levels of explicit prejudice and intergroup anxiety post-intervention were found to be lower for participants exposed to the BPD disclosure. The sample size for a follow-up study 1-week post-intervention was not large enough for definitive conclusions, but exploratory analyses suggested that participants exposed to the disclosure video thought about mental health more positively, and that lowered prejudice levels from the intervention withstood time.

These findings expand our parasocial understanding in an exciting way, supporting the parasocial contact hypothesis^12^ and demonstrating further that one-way disclosure is sufficient to induce relationship strength, even in alternative contexts that centre around a single experience such as mental health (as opposed to more general and mutual self-disclosure)^26^.

Implicit measures of prejudice did not significantly differ within subject pre- and post-intervention. Internal motivation does not always translate into external action^36^ and so this null finding may not be too underwhelming, as explicit prejudice reduction is said to be more valuable for societal improvement^36^. A possible reason for the unaltered implicit prejudice could simply be that implicit prejudice values are harder to manipulate, as beliefs are extremely resistant to change^37^. Alternatively, the IAT (albeit a common tool in implicit prejudice research) may not be a suitable method to detect such change^38^. Alternative implicit measures, such as electroencephalography, heart rate, and galvanic skin response, may be more accurate at measuring prejudice on an implicit dimension. Future studies could explore such alternatives to understand whether affecting implicit prejudice is possible.

Even though our PSR manipulation failed to create stronger PSRs for those in the same-creator condition than those in the different-creator group, our study suggests that, in order to reduce prejudice, no previous PSR-building is required before a parasocial target discloses about their marginalised experience. Hence, an existing parasocial relationship is not necessarily required in prejudice interventions, reflecting an even greater capacity for parasocial targets to create prosocial change with anyone who encounters them, even just once. With over 37 million creators on YouTube and 500 hours of video content being uploaded every minute^39^, if outgroup parasocial targets candidly sharing their experiences can lower prejudice, the passively formed PSRs that viewers are already creating can lead to a more accepting and cohesive society. However, future research may benefit from incorporating an additional condition for which no PSR is created (e.g., with an automated neutral voice). A successful manipulation in this way would enable exploration of interaction effects where varying degrees of initial prejudice may affect prejudice levels following the intervention.

Future studies could also replicate this experiment format with different minority experiences, such as disclosure stimuli on the personal experiences of transgender people, or people of ethnic minorities. This may establish whether this intervention is successful in alternative intergroup contexts also, and whether additional parasocial benefits can be gained by minority communities specifically. For example, past research has shown that LGBT+ individuals specifically benefit from parasocial interaction, particularly those with low social support^40^, and particularly as LGB adolescents seem to compensate for an absence of in-person LGB peers, with LGB content creators^41^.

A further limitation to consider is the limited follow-up study sample size. One week after the intervention, lower prejudice levels were maintained within experimental conditions, and participants from the treatment groups had also thought about supporting mental health initiatives more than participants from the control group. No participants reported anti-support behaviours, but longitudinal conclusions are made conservatively due to low sample sizes. Future research should consider examining larger cohorts of participants over longer periods of time, and may also wish to consider whether participants identify as ingroup or outgroup for measures.

Creators from underrepresented backgrounds sharing their content online supports a new scale of global citizenship, empowerment, and voice, and can provide additional benefits to traditional coverage of stigmatised communities by providing more authentic portrayals to sensationalised traditional media^23,41^. Future research could examine whether marginalised community members gain different levels and types of emotional need fulfilment from ingroup parasocial targets when compared to outgroup parasocial targets, and whether it is easier to develop greater parasocial strength with a target who shares race, disability, or identity. This seems likely, as identification with social groups supported greater perceived need fulfilment in prior bidirectional research^42^. It would also be interesting to understand whether the influence of parasocial targets can be harnessed more effectively if the target is also an ingroup member. For example, Black communities are known to have greater hesitancy towards medical interventions due to historical mistreatment of people of colour which still percolates our societies^43^. It could therefore be valuable to investigate whether PSRs developed with Black creators are more likely to encourage Black people to receive essential medical care than creators who are not Black.

Whilst our PSR intervention worked to reduce prejudice to people with mental health issues, this stigma is not necessarily apparent straight away. Whether prejudice towards non-concealable characteristics, such as ethnicity, can also be reduced through PSR prejudice interventions remains an open question^44^. For people who may be prejudiced, developing a PSR before disclosure may encourage them to experience the disclosure about the concealable and stigmatised identity in due course, providing an opportunity for parasocial contact to reduce prejudice. However, if somebody is prejudiced against people of colour, it seems unlikely that a black person sharing their experiences will work as a prejudice intervention in real life, particularly because those with prejudice may not be willing to listen to them in the first place,. As the colour of one’s skin is not concealable in the same way a mental health issue may be, it seems less likely that PSR strength would develop. An interesting future direction could therefore be to consider an experimental design that explores whether PSR prejudice interventions are successful for non-concealable stigmatised identities also, perhaps using non-visual stimuli (e.g., podcasts, or radio) to research this aspect.

As PSRs already exist and influence society, it must be noted that parasocial targets may also have the capacity to communicate negative outgroup portrayal, intentionally or otherwise. Unfortunately, media is all too often harnessed by political and corporate elites to shape public agenda^45^, leading to unfavourable outgroup portrayal. For example, disproportionate casting of ethnic minorities as villains^46^ reinforces negative stereotypes and stigma, causing individuals to avoid personal contact with outgroups^47^. As technology today reduces the gatekeeping of large audiences and allows anybody to develop audiences online, the potential for antisocial influence should not be dismissed. However, such gatekeeping absence has also allowed for more realistic and positive representation than ever before, providing a unique pathway to prosocial impact. A limitation of the existing research is that negative influence from PSRs were not thoroughly examined, and so future research could examine and compare the effectiveness of prosocial and antisocial encouragement further.

We would also like to note potential limitations in our study sample and generalisability of findings. We limited the age range of participants to match that of our creators’ audiences (i.e., 18–35 years of age) and, due to the nature of the stimuli, to participants in English-speaking countries. Within these parameters, our effects are shown across a range of genders and ages. Adults under 35 years of age are the biggest group of YouTube users^22,48^. However, future studies may wish to replicate existing study designs across populations in other countries, and with different creators who broadcast content around a range of topics and values, as opposed to only liberal content surrounding ethnicity, LGBT+ identity, and mental health.

Finally, prejudice intervention studies have been criticised for producing small effects when bigger interventions are needed for an issue as invasive and pertinent as prejudice^49^. Whilst large-impact interventions should continue to be explored, there is value in recognising that millions of PSRs are passively created in everyday activities and have the potential to consistently portray outgroup experiences in ways that reduce prejudice, albeit on a smaller level. While PSRs may not be the “stronger medicine” required for radical societal change, they can be spread over time and are easily accessible. Combined with the speed in which PSRs can be formed, they present a more time-efficient, cost-efficient, and largely scalable remedy, compared to interventions that require face-to-face interaction.

## Data Availability

The datasets generated and analysed during the current study, as well as the code needed to replicate our results, are available in the Open Science Framework (OSF) repository, https://osf.io/nsrv5/.
